# Amphiphilic Nanocarrier Systems for Curcumin Delivery in Neurodegenerative Disorders

**DOI:** 10.3390/medicines5040126

**Published:** 2018-11-23

**Authors:** Miora Rakotoarisoa, Angelina Angelova

**Affiliations:** Institut Galien Paris-Sud CNRS UMR 8612, LabEx LERMIT, Univ Paris-Sud, Univ Paris-Saclay, F-92296 Châtenay-Malabry, France; miora.rakotoarisoa@u-psud.fr

**Keywords:** curcumin, lipid nanoparticles, liquid crystalline carriers, nanomedicines, neuroprotection, antioxidant

## Abstract

Neurodegenerative diseases have become a major challenge for public health because of their incurable status. Soft nanotechnology provides potential for slowing down the progression of neurodegenerative disorders by using innovative formulations of neuroprotective antioxidants like curcumin, resveratrol, vitamin E, rosmarinic acid, 7,8-dihydroxyflavone, coenzyme Q10, and fish oil. Curcumin is a natural, liposoluble compound, which is of considerable interest for nanomedicine development in combination therapies. The neuroprotective effects of combination treatments can involve restorative mechanisms against oxidative stress, mitochondrial dysfunction, inflammation, and protein aggregation. Despite the anti-amyloid and anti-tau potential of curcumin and its neurogenesis-stimulating properties, the utilization of this antioxidant as a drug in neuroregenerative therapies has huge limitations due to its poor water solubility, physico-chemical instability, and low oral bioavailability. We highlight the developments of soft lipid- and polymer-based delivery carriers of curcumin, which help improve the drug solubility and stability. We specifically focus on amphiphilic liquid crystalline nanocarriers (cubosome, hexosome, spongosome, and liposome particles) for the encapsulation of curcumin with the purpose of halting the progressive neuronal loss in Alzheimer’s, Parkinson’s, and Huntington’s diseases and amyotrophic lateral sclerosis (ALS).

## 1. Introduction

Neurodegenerative diseases (Alzheimer’s disease (AD), Parkinson’s disease (PD), Huntington disease (HD), and amyotrophic lateral sclerosis (ALS)) are disabling chronic disorders characterized by the progressive loss of neurons in different areas of the central nervous system. Neuronal cell death leads to cognitive, behavioral, sensory, and motor dysfunctions [1,2,3,4,5,6,7,8,9,10,11,12,13]. Currently, age-related neuronal diseases have higher incidences because of increasing life expectancies. Neurodegenerative disorders are caused by multiple factors, such as the accumulation of misfolded proteins, the depletion of endogenous antioxidant enzyme activity, mitochondrial dysfunction, and the deficiency of neurotrophin brain-derived neurotrophic factor (BDNF), neuro-inflammation, as well as various genetic mutations [14,15,16,17,18,19,20,21,22,23,24,25,26,27,28,29,30].

In recent years, several studies have shown that curcumin is a safe natural compound which may prevent the deleterious effects of risk factors causing brain damage as well as slowing down the progressive neuronal loss via different pathways [26,27,28,29,30,31,32,33,34,35,36,37,38,39,40,41,42,43,44,45,46,47,48,49,50]. However, clinical studies performed with AD patients with various degrees of progression have reported poor results on the AD symptoms following curcumin treatment [51,52,53,54]. This did not allow firm conclusions about the therapeutic or neuroprotective potential of curcumin to be drawn. The obstacles for curcumin utilization as a drug originate from its limited water solubility, poor physicochemical stability, high-grade metabolism, and low plasma concentrations [36,53,54,55]. The development of nanoparticulate delivery systems for curcumin has attracted scientific interest in order to improve its bioavailability and stability as a drug compound [56,57,58,59,60,61,62,63,64,65]. Curcumin administration to neurodegenerative disease models by nanoparticles has been realized using liposomes, solid lipid nanoparticles, and polymeric particles. Delivery by other carriers such as amphiphilic proteins, e.g., casein, is also possible, but has not been examined as a means of transporting curcumin across the BBB towards neuro-regeneration.

In this review, we briefly summarize the in vitro and the in vivo evaluations of curcumin, which are linked to multiple risk factors and the multi-target mechanisms of neurodegenerative diseases, and discuss the reported clinical investigations of varying efficacy in humans. Then, we highlight the variety of amphiphilic curcumin-loaded nanocarriers including liposomes, liquid crystalline nanoparticles (cubosomes, hexosomes, and spongosomes), solid lipid nanoparticles, micelles, and polymeric nanoparticles as potential nanomedicine formulations in regeneration therapies of the major neurological disorders.

## 2. Risk Factors for Neurodegenerative Disorders

Alzheimer’s disease (AD) is the most common cause of dementia. It currently affects about 10% of the world’s population over 60–65 years of age, and about 50% over 85 years of age. More than 30 million people may expect to be affected by AD during the next 20 years due to the increasing lifespan of the world population [1,2]. Major pathological features of AD include the accumulation of extracellular amyloid plaques and fibrils, intracellular neurofibrillary tangles, and disruption of the cholinergic transmission, including reduced acetylcholine levels in the basal forebrain (Table 1). The most common symptom is the short-term memory loss, i.e., difficulty in remembering recent events [2,3,4,5]. Other symptoms include disorientation, mood, language, and behavioral issues, and loss of motivation, depending on the progression of the disease. The treatments of AD have employed acetylcholinesterase inhibitors (tacrine, rivastigmine, galantamine, and donepezil) to overcome the decrease of the ACh levels as a result of the death of cholinergic neurons. The NMDA receptor antagonist (memantine) acts by inhibiting the overstimulation by glutamate, which can cause cell death. Atypical antipsychotics have modest efficacy in reducing the aggression and psychosis of AD patients. These medications provide little benefit, and provoke various adverse effects [6,7]. 

The second most common disorder, Parkinson’s disease (PD), affects more than 1% of the population over 60 years of age and 5% over 85. PD is characterized by progressive impairments in locomotive ability such as tremor, rigidity, and bradykinesia. These symptoms are attributed to the loss of dopaminergic neurons in the substantia nigra and the formation of Lewy bodies in the brain [8,9]. Treatments are symptomatic and aim at boosting the depleted levels of dopamine (Table 1). The most used drug is levodopa. Dopamine agonists are used when the treatment by levodopa becomes less efficient. The inhibitors of MAO-B and COMT (safinamide, selegiline, rasagiline, and tolcapone) are used to inhibit the activity of the enzymes which degrade dopamine. These medications become less effective as the neurons are continuously lost during disease progression. At the same time, they produce complications marked by the involuntary movements of the patients [8,9].

Huntington disease (HD) is a rare disease which affects about 1/10,000 people (usually between 30 to 50 years of age) in the United States and 1/18,000 people in Europe. It is a poly-glutamine (PolyQ) autosomal genetic disorder characterized by impairments of cognitive, psychiatric, and motor functions [10]. The hallmark of the HD pathology is the abnormal accumulation of misfolded mutated huntingtin protein (mHTT) as intracellular aggregates. They cause selective neuronal loss, primarily in the cortex and in the medium spiny neurons of striatum. Symptoms develop from a general lack of coordination to apparent uncoordinated, jerky body movements [11]. The physical abilities of the patients gradually worsen until coordinated movement becomes difficult. There is no effective cure available to HD (Table 1). The only approved medication, tetrabenazine, and other tested drugs (neuroleptics and antipsychotics) help to reduce chorea and psychiatric symptoms.

Amyotrophic lateral sclerosis (ALS) is a severe debilitating disease caused by motors neurons degeneration in the brain and the spinal cord. It is generally characterized by progressive paralysis starting at the limbs and ultimately leading to death caused by respiratory failure within 3 to 5 years after the onset of the symptoms. There is no cure for ALS (Table 1). The approved medication, riluzole, may extend life by just a few months [12,13].

The pathological characteristics, genetic factors, clinical symptoms, and actual medications of these diseases are summarized in Table 1. It should be emphasized that the existing therapeutic approaches do not exert disease-modifying effects on the neurodegeneration. The associated economic and societal challenges lead to a growing public health burden.

**Figure 1:**
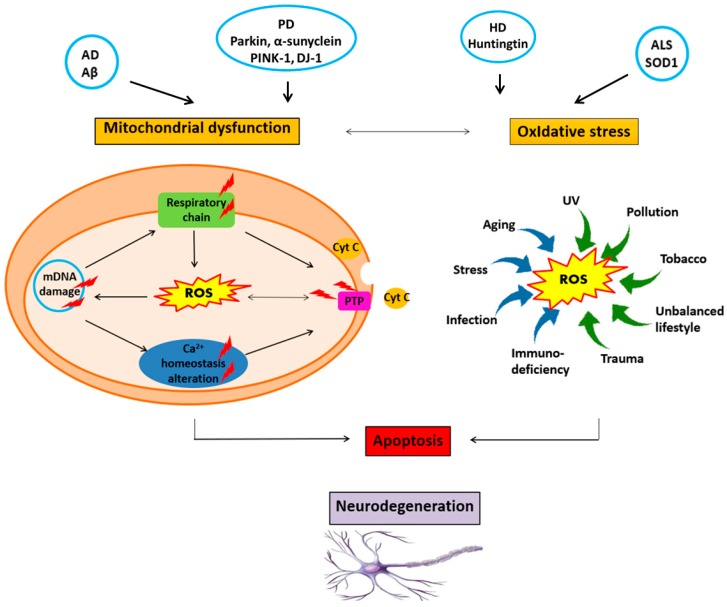
Neurodegeneration is triggered and boosted by a vicious circle involving neurotoxic protein accumulation, oxidative stress, mitochondrial damage, DNA damage, and impairment of the calcium (Ca^2+^) homeostasis, neurotrophin deficiency, neuroinflammation, genetic, and environmental factors.

Although the etiology and the pathological mechanism of the neurodegenerative diseases are not completely understood, it has been established that the progressive loss of neurons results from the combination of multiple factors (Figure 1). First, genetic factors are involved in the appearance of misfolded amyloid-Aβ protein and other misfolded mutant forms like hyperphosphorylated Tau (p-Tau) and Huntingtin proteins [3,4,5,10,14]. All these mutated proteins aggregate and form deposits. The resulting aggregates can be toxic, and can affect the intracellular organelles such as mitochondria [14,21,25,27]. The disruption of the mitochondrial membrane causes neuronal cell death [25,27].

Second, neurotrophic factors deficiency has been reported in the severe neurodegenerative disorders [11,13,29,30,31,32,33]. Neurotrophins regulate the neuronal survival, differentiation, growth, and regeneration, as well as the synaptic plasticity. Studies have shown that the levels of brain derived neurotrophic factor (BDNF) and its tropomyosin kinase B (TrkB) receptor are decreased in the hippocampus and the cerebral cortex at the beginning of the Alzheimer’s disease [11]. In addition, the administration of BDNF mimetics into transgenic mouse models of AD has enhanced learning and memory capacities [31]. 

Third, oxidative stress is the most common feature of neurodegenerative diseases [15,16,17,18,19,20]. Reactive Oxygen Species (ROS) such as superoxide anions, hydroxyl radicals, and hydrogen peroxide (H_2_O_2_) are produced by the mitochondrial transport chain, the endoplasmic reticulum, the Krebs cycle, and the plasma membrane involving the superoxide-generating NADPH oxidase (NOX) macromolecular complex [17]. Oxidative stress occurs under environmental factors and when the generation of ROS exceeds the natural antioxidant defenses of the cell (promoted by superoxide dismutase, catalase, glutathione peroxidase, carotenoids, and vitamins E or C) [15,16,17,18,19,20]. ROS accumulation attacks proteins, nucleic acids, and membrane lipids, and thus, causes impairments of the neuronal cell functions and integrity [18,19,20]. Mitochondrial lesions are also mediated by ROS. This leads to the alteration of the neuronal cell bioenergetics, the disruption of the calcium (Ca^2+^) homeostasis, or the activation of the mitochondrial permeability transition pore (mPTP). Thus, a vicious cycle is formed (Figure 1), which amplifies the cellular dysfunction and triggers neurodegeneration [17,18,19,20,21,22,23,24,25,26]. 

Fourth, neuro-inflammation is a crucial factor that favors neurodegenerative disease development. Several inflammatory markers (such as chemokines, cytokines, or proteins in the acute phase) are upregulated and cause inflammation. In fact, elevated levels of the inflammatory markers have been found during the progression of the neurodegenerative diseases [27,28].

## 3. Curcumin Potential for Neuroprotection against Neurodegenerative Diseases

**Figure 2:**
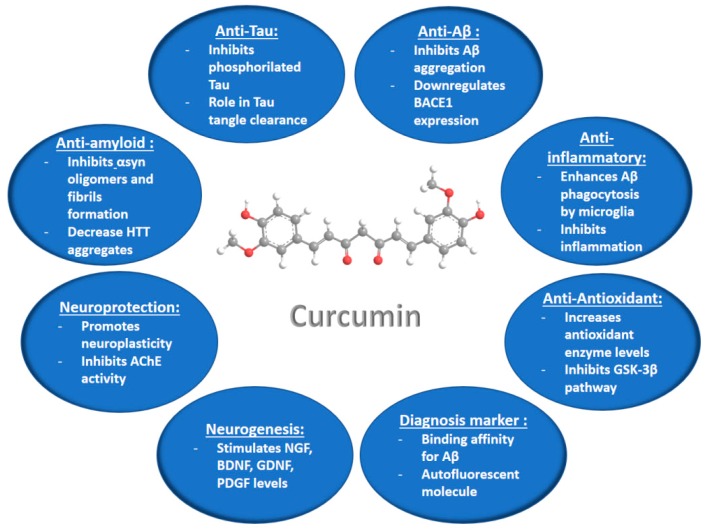
Summary of the curcumin activities and suggested mechanisms of action, which can be exploited for treatment of neurodegenerative diseases (according to information from ref. [36]).

Curcumin is a hydrophobic polyphenolic substance (Figure 2) produced in the root of the plant *Curcuma Longa* L. This antioxidant compound is extensively marketed worldwide as a nutraceutical in various preparations, because it has a very safe nutraceutical profile with low side effects. Curcumin has been reported to be well tolerated at doses up to 8 g per day over short periods in humans [32]. Research on the pharmacological activities of curcumin has attracted strong attention in relation to its multiple actions of therapeutic interest, e.g., the anti-inflammatory, antioxidant, antiviral, antibacterial, antifungal, and antitumor activities. These activities appear to be dose-dependent [33]. 

### 3.1. In Vitro and In Vivo Studies of Curcumin Properties in Neurodegenerative Disease Models

The neuroprotective potential of curcumin (Figure 2) and its antioxidant, anti-inflammatory, and amyloid Aβ binding properties have been highlighted in in vitro and in vivo investigations of different neurodegenerative disease models [33,34,35,36,37,38,39,40,41,42]. Curcumin has been found to increase the levels of glutathione (GSH) and malondialdehyde (MDA), as well as the antioxidant enzyme [superoxide dismutase (SOD), glutathione peroxidase (GPx), glutathione reductase (GR), and catalase (CAT)] activities in the rat brain, thus preventing the stress-induced oxidative damage of brain [37,39]. The anti-inflammatory properties of curcumin have been characterized by the inhibition of the inflammatory chemokines, by increased levels of the anti-inflammatory cytokines, and by enhanced expression levels of induced nitric oxide synthase (iNOS) and the transcription factor NF-Kb [39]. Curcumin has been shown to prevent the fibrillation of α-synuclein at the earliest stage of the aggregation process, as well as the fibrillation of the globular protein hen egg-white lysozyme (HEWL) [40]. Both proteins are known to form amyloid-like fibrils. These results have suggested that curcumin might be a potential therapeutic agent for preventing protein aggregation in Alzheimer’s, and Parkinson’s diseases [40]. Recent in vitro and in vivo investigations of curcumin’s activities in neurodegenerative disease models [41,42,43,44,45,46,47,48,49,50] are summarized in Table 2.

### 3.2. Clinical Trials and Curcumin Limits

A serious obstacle to the pharmaceutical application of curcumin has arisen from its limited water-solubility and low bioavailability. In addition, this compound is chemically instable, which may cause a loss of biological activities. The failure of free curcumin in clinical trials is likely due to its limited bioavailability. For instance, curcumin has been delivered in doses between 1 and 4 g/per day as capsules or as powder mixed with food in trials for treatment of Alzheimer’s disease patients. The performed 6-month treatment study found no differences in the Aβ-amyloid levels between the treatment groups or in the Mini Mental State Examination (MMSE) scores [51]. Similarly, oral curcumin in a 24-week, randomized, double blind, and placebo-controlled study for AD treatment has shown no detectable differences in the measured biomarkers from the different treatment groups [52]. A clinical study with three single cases of patients receiving curcumin (100 mg/day) reported that only one patient increased his MMSE score from 12/30 to 17/30 after 12 weeks of treatment (improved calculation, concentration, transcription of the figure, and spontaneous writing). Two of the patients were on donepezil treatment before starting the curcumin trial [53]. Based on all performed trials with AD patients, it was, however, difficult to conclude if curcumin has positive effects on the AD symptoms [54].

In fact, the major fraction (35–89%) of orally-administered curcumin can be lost due to its low bioavailability. The intestinal mucosa and mucus form a physical barrier to curcumin adsorption. The drug cannot reach the circulation in a bioactive form as it undergoes reduction and metabolism/conjugation in the liver. Reductases enzymatically reduce curcumin to dihydrocurcumin, tetrahydrocurcumin, and hexahydrocurcumin. Furthermore, curcumin may be conjugated to sulfates and glucuronides [55,56,57]. Thus, most of the circulating curcumin is in a conjugated form. 

The necessity of the development of a delivery system for the protection of curcumin from rapid metabolism and for the improvement of its bioavailability has become evident [58]. A randomized, double-blind, placebo-controlled clinical trial examined the acute administration (effects 1 h and 3 h after a single dose application), chronic (4 weeks) administration, and acute-on-chronic (1 h or 3 h after a single dose followed by a chronic treatment) effects of solid-lipid-nanoparticle (SLNP) loaded by curcumin. The results of the SLNP formulation of curcumin (400 mg in capsules Longvida^®^) on cognitive function, mood, and blood biomarkers were obtained with 60 healthy adults (aged 60–85). SLNP-loaded curcumin significantly improved the performance in sustained attention and working memory tasks one hour after its administration (as compared to placebo). Working memory and mood (general fatigue and change in the calmness state, contentedness, and fatigue induced by psychological stress) were essentially improved following chronic treatment. A significant acute-on-chronic treatment effect on alertness and contentedness was also observed [59].

## 4. Nanocarrier-Mediated Curcumin Delivery

Nanotechnology for nanomedicine development employs functional materials with appropriate nanoscale organization that can interact with biological systems and induce desired physiological responses while minimizing undesirable side effects [60]. Nanotechnology-based delivery systems can influence drug capacity to cross the biological barriers (e.g., the BBB) and reach the targeted brain regions [58,59,60,61]. Therefore, nanocarriers are promising for the development of personalized medicines for the treatment of neurological disorders [62,63,64,65,66,67].

**Figure 3:**
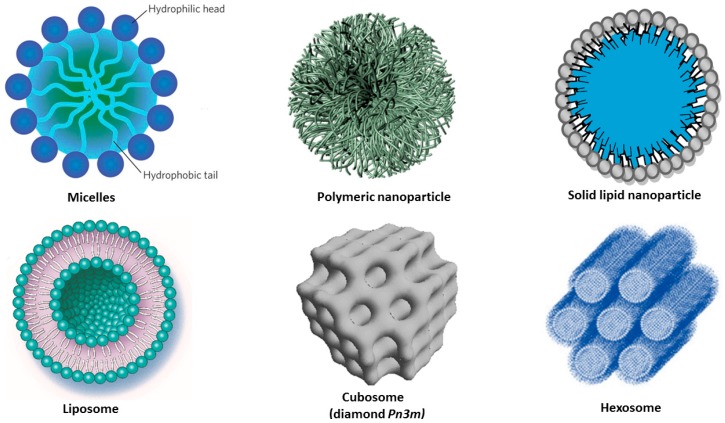
Schematic presentation of amphiphilic nanocarriers enabling the encapsulation and protection of hydrophobic and hydrophilic molecules of therapeutic significance.

Lipid-based nanoparticles, including solid lipid nanoparticles (SLNPs), nanostructured lipid carriers (NLC), liposomes and liquid crystalline nanocarriers (LCN), as well as polymer-based nanoparticles (Figure 3), have been developed to overcome the poor solubility, stability, and bioavailability of curcumin, and to promote its utilization as a drug in disease treatments [68,69,70,71,72,73,74,75,76,77,78,79,80,81,82,83,84,85,86,87,88,89,90,91,92,93,94,95,96,97,98,99,100,101,102,103,104,105,106,107,108,109,110,111,112,113,114,115,116,117,118,119,120,121,122,123,124]. 

Lipid-based nanoparticles have the advantage of being the least toxic carriers for in vivo applications. The lipids used to prepare biocompatible and biodegradable nanoparticles are usually naturally-occurring molecules with low acute and chronic toxicity. In the case of polymeric nanoparticles, the in vivo degradation of the polymer matrices might cause toxic effects [94]. The biocompatibility and the physico-chemical diversity of lipids and their capacity to enhance the oral bioavailability of drugs have made this kind of nanocarriers very attractive systems for drug delivery. As a matter of fact, lipid-based formulations may positively influence drug absorption in several ways, e.g., by influencing the solubilization properties, preventing the drug precipitation upon intestinal dilution, increasing the membrane permeability, inhibiting the efflux transporters, reducing the CYP enzymes, or enhancing the lymphatic transport [94,122]. 

Among the lipid-based nanoparticles, SLNPs have been intensively developed because they combine the advantages of different carrier systems like liposomes and polymeric particles. Similarly to liposomes, SLNPs are composed of physiologically-biocompatible excipients (lipids and fatty acids). In the same way to polymeric NPs, their solid matrix core can efficiently protect the loaded active pharmaceutical ingredient against chemical degradation under the harsh conditions of biological milieux. Therefore, SLNPs provide controlled release profiles of the encapsulated drugs [95]. 

In addition to the above advantages, liposomes can encapsulate and transport both lipophilic and hydrophilic drugs. They have a high degree of similarity to cell membranes in terms of lipid composition and organization, which facilitates the bioavailability of the pharmaceutical compounds [102]. Liquid crystalline nanocarriers (LCN) such as cubosomes and hexosomes (Figure 3) involve multiple compartments for encapsulation of either lipophilic or hydrophilic drugs. They display structural advantages which enable high encapsulation efficacy for molecules of various sizes and hydrophilicity [113,116]. LCNs are formed by self-assembly of lyotropic lipids such as unsaturated monoglycerides, phospholipids, glycolipids, and other amphiphilic molecules. For example, monoolein, which is a nontoxic, biodegradable, and biocompatible lipid, is classified as a GRAS (Generally Recognized As Safe), and ω-3 polyunsaturated fatty acids (n-3 PUFA) have been shown to be highly beneficial in various disease models of neurodegeneration [123,124].

In the following section, we summarize recently reported works on curcumin delivery to in vitro and in vivo models of neurodegenerative diseases.

### 4.1. Curcumin Delivery by Polymeric Nanoparticles

Polymeric nanoparticles of a biocompatible and biodegradable nature are of essential interest as drug delivery nanocarriers. The release of the encapsulated drug can be modulated by altering the polymer composition and amphiphilicity. Poly(lactic-co-glycolic acid) (PLGA) is one of the most commonly-used biodegradable synthetic polymers. It is a FDA- (US) and EMA-approved platform for the delivery of drugs to humans. PLGA-derived nanoparticles have been successfully used for the encapsulation of different hydrophobic compounds (such as curcumin) by nanoprecipitation or by single emulsion techniques [71]. Hydrophilic molecules can be encapsulated by means of double emulsions or by two-step nanoprecipitation methods. On the other site, polymeric micelles have been studied towards site-specific drug delivery [68,69,70]. 

Micelles are formed by amphiphilic macromolecules, which self-assemble into nano-sized (10–100 nm in diameter) core/shell structures in excess aqueous media (Figure 3, top). The core-shell organization facilitates the incorporation of curcumin inside the hydrophobic core, while the water solubility of the nanocarriers is ensured by their hydrophilic corona [68]. Amphiphilic co-polymers self-assemble into micelles in aqueous solutions due to the hydrophobic interactions among their water-insoluble segments. Curcumin-loaded polymeric micelles have received attention due to various features (Table 3), like (i) the enhanced solubility of the drug; (ii) the sustained CU release profile; and (iii) the small size of the PEG-decorated carriers (<200 nm), which stabilizes them in biological fluids [64,65,66]. Micelles formed by the PLGA-PEG-PLGA synthetic copolymer have shown potential in modifying the pharmacokinetics and tissue distribution of curcumin. Evaluation of pharmacokinetics and biodistribution has demonstrated a prolonged half-life of the CU-micelles and a more efficient drug delivery to brain areas as compared to the carrier-free CU administration [71].

### 4.2. Curcumin Delivery by Lipid Nanoparticles

#### 4.2.1. Solid Lipid Nanoparticles (SLNPs) and Nanostructured Lipid Carriers (NLCs)

Solid lipid nanoparticles (SLNPs) are submicron colloidal lipid carriers (from 50 nm to 1000 nm in diameter) which maintain a solid, spherical shape at room temperature. They possess a solid lipid core matrix stabilized by emulsifiers that can solubilize lipophilic molecules. The CU-SLNPs are usually small, ranging from 100 to 300 nm in diameter. The total drug content can reach up to 92% when the SLNPs are manufactured using the micro-emulsification technique [87]. In an experimental rat model of cerebral ischemic reperfusion injury, animals fed with CU-loaded SLNPs have had a 90% improvement in their cognitive function along with a 52% inhibition of the acetyl cholinesterase activity [88]. The investigated formula has been shown to increase the levels of superoxide dismutase (SOD), catalase (CAT), glutathione (GSH), and the activities of mitochondrial enzymes, while decreasing the lipid peroxidation and the peroxynitrite levels. Furthermore, this formulation showed a 16.4 to 30-fold improvement in the bioavailability of CU in the brain upon oral and intravenous (IV) administrations, respectively [88]. The product Longvida^®^ (Verdure Sciences Inc.) is a SLNP-formulation of curcumin which can yield from 0.1 to 0.2 μM plasma levels of CU with associated 1–2 μM brain levels of free CU in animals [89,90,91]. This formula was later optimized as “lipidated Cur”, which can yield more than 5 μM CU in mouse brain [93]. Other formulations of CU-loaded SLNPs, tested in Alzheimer’s disease models, are outlined in Table 4.

Nanostructured lipid carriers (NLC) are referred to as the “second generation” of SLNPs. NLCs are composed of mixtures of sterically different amphiphilic molecules. Often, mixtures of liquid-phase lipids and solid-phase lipids yield matrices with imperfections, which may incorporate increased quantities of drug molecules as compared to the SLNPs. Despite of the presence of the liquid-phase lipid, the NLC matrix appears to be in a solid state at room and body temperatures. The solid state is controlled by the fraction of the included liquid-phase lipid [94]. Sadegh-Malvajerd et al. have reported an enhanced entrapment efficiency of curcumin in NLCs (94% ± 0.74) as compared to SLNPs (82% ± 0.49). The pharmacokinetic studies, performed after intravenous (IV) administration of 4 mg/kg dose of curcumin in rats, have indicated that the amount of curcumin available in the brain was significantly higher for curcumin-loaded NLCs (AUC_0–t_ = 505.76 ng/g h) as compared to free curcumin (AUC_0–t_ = 0.00 ng/g h) and curcumin-loaded SLNs (AUC_0–t_ = 116.31 ng/g h) (*P* < 0.005) [95]. The outcomes of other recent investigations of CU-loaded NLCs in models of neurodegenerative diseases are summarized in Table 4.

#### 4.2.2. Liposomes

Liposomes are lipid bilayer-based, self-assembled, closed colloidal structures, typically 25 nm to 5 μm in diameter [97]. They usually have a spherical shape comprising an aqueous core surrounded by a hydrophobic lipid membrane (Figure 3). The lipid bilayer can be loaded with hydrophobic or amphiphilic molecules, whereas the hydrophilic molecules can be encapsulated in the aqueous reservoir of the liposomes. Often, liposomes are composed of phospholipids (e.g., phosphatidylcholines) or mixtures of phospholipids with co-lipids. Various liposome architectures can form depending on the preparation methods; for instance, multilamellar vesicles (MLV, involving a stack of several lipid bilayers), small unilamellar vesicles (SUV, constituted by a single lipid bilayer), large unilamellar vesicles (LUV), tubular vesicles, and cochleate vesicles. 

Curcumin encapsulated in liposomes has been proven to be a safe formulation, which enhances the CU solubility and its cellular uptake [97,98,99,100,101]. Liposomes deliver CU into the cells via membrane fusion or endocytosis process. Liposomal formulations with a PEG surface coating provide a longer circulation time for the encapsulated drug. Biomolecular ligands can be anchored to the liposome surface in order to enhance the receptor targeting capacity, and hence, the permeability across the brain-blood barrier (BBB) [102,103]. The outcomes of the investigated CU-loaded liposomes in models of neurodegenerative diseases are summarized in Table 4. 

#### 4.2.3. Liquid Crystalline Nanoparticles (LCNPs) with Internal Structure

Liquid crystalline nanoparticles (LCNPs) are self-assembled architectures of lyotropic lipids, co-lipids (surfactants or oils), and water. They are typically formed upon dispersion and fragmentation of bulk lyotropic liquid crystalline phases (e.g., bicontinuous cubic, sponge, or inverted hexagonal phases) [104,105,106]. The amphiphilic molecules spontaneously organize into compartments with hydrophobic and hydrophilic domains (Figure 3), which can encapsulate lipophilic or hydrosoluble guest compounds. The structures formed by this self-assembly process are thermodynamically stable. The initial liquid crystalline phases are usually viscous and have a short-range order in comparison to solids, but long-range order in comparison to liquids. A typical example of a lyotropic liquid crystalline phase is the inverted bicontinuous cubic phase formed upon mixing of unsaturated monoglyceride lipids with water [105]. Cubosomes are produced upon dispersion of the bicontinuous cubic liquid crystalline phases in excess aqueous medium. Their periodic structures comprise folded bicontinuous lipid bilayer membranes and periodic networks of aqueous channels (Figure 3). The latter enable high encapsulation capacity for hydrophilic guest macromolecules [106,107,108,109,110,111]. Lipid nanocarriers of liquid crystalline internal structures have received considerable attention as delivery vehicles through the BBB [111,112]. 

Curcumin has been successfully entrapped into monolein-based LCNPs with almost 100% encapsulation efficiency [113]. LCNPs dispersion was very stable in terms of nanocarrier sizes and surface charge upon storage. LCNPs were efficiently taken up by cultures cells following the sustained release of curcumin. In addition, they provided inhibition of the cell proliferation and apoptosis induction in an anticancer study [113]. A recent investigation of an inverse hexagonal (H_II_) liquid crystalline phase encapsulating curcumin has demonstrated that the release of curcumin was a concentration-diffusion controlled process in the early stages, whereas multiple diffusion mechanisms coexisted in the later stages of drug release. Radical scavenging experiments have shown that curcumin-loaded LCNPs exert the desired antioxidant activity [114]. Thus, curcumin-loaded LCNPs may be promising for neurodegenerative disease treatments using sustained-release nanoformulations for combination therapies [115,116]. Further results obtained with lipid-based LCNPs in models of neurodegenerative disease are presented in Table 4.

## 5. Conclusions

The naturally occurring compound curcumin is increasingly studied in neurodegenerative disease models due to its neurogenesis-stimulating properties and its anti-amyloid and anti-tau potential. Nanotechnology-based delivery systems of curcumin have been developed with the purpose of improving its solubility, stability, and bioavailability in potential treatment strategies of neurodegenerative disorders. We summarized recent advances in research on safe liquid crystalline lipid-based nanocarriers (cubosome, spongosome, hexosome, and liposome particles) and solid lipid nanoparticles, as well as on selected biodegradable polymer-based nanocarriers. The emphasis is given on the observed biological outcomes of the curcumin nanoformulations in in vitro and in vivo models of the multifactor neurodegenerative diseases (AD, PD, HD and ALS). Despite the difficulty of overcoming biological barriers, promising results on the enhancement of the permeability of the BBB and receptor-mediated delivery across the BBB have been reported with liposome and cubosome nanocarriers. Further investigations will be required in order to understand the involved mechanisms of action of curcumin nanoformulations in the proposed neurodegenerative disease models, and to optimize the delivery systems and strategies towards translation into clinics. 

## Figures and Tables

**Table 1:** Pathological characteristics, genetic factors and clinical symptoms of Alzheimer’s disease (AD), Parkinson’s disease (PD), Huntington’s disease (HD), and amyotrophic lateral sclerosis (ALS) [1,2,3,4,5,6,7,8,9,10,11,12,13,14,15,16,17,18,19,20,21,22,23,24,25,26,27,28,29,30].

Diseases	Characteristics	Genetics factors	Symptoms	Actual treatments
AD	Senile plaques from extracellular amyloid-Aβ accumulation, Intracellular neurofibrillary tangles, Tau protein aggregation, Irreversible neuronal loss, Brain atrophy	Inherited form (70% of patients): mutations of APP, PSEN1 or PSEN2. Sporadic form (30%): presence of ApoE4 allele in the ApoE gene	Progressive memory loss, Decision judgement loss, Autonomy loss	Anticholinergics (tacrine, rivastigmine, galantamine and donepezil), Memantine, Antipsychotics, NSAIDs
PD	α-Synucleinopathy, Presence of Lewy bodies, Degeneration of dopaminergic neurons in the substance nigra of the brain, Dopamine deficiency	Gene mutations: α-synuclein SNCA, Parkin PRKN, PARK7, PINK1, LRRK2, GBA, DJ-1, VPS35, EIF4G1, DNAJC13 and CHCHD2	Hypokinesia, Bradykinesia, Rigidity, Postural instability, Neuropsychiatric disturbances	Levodopa, Dopamine agonists, MAO-B inhibitors, COMT inhibitors, Anticholinergics
HD	Accumulation of mutant Huntingtin protein in the brain	Expansion of CAG trinucleotide in Huntingtin gene (HTT)	Chorea, Cognitive and neuropsychiatric disorders	Tetrabenazine, Neuroleptics, Antipsychotics
ALS	Progressive degeneration of motor neurons	Sporadic form: 90% of patients Inherited form: 10% Mutations of SOD1, TARDBP, FUS, UBQLN2, OPTN, and C9ORF72 genes	Spasms, Muscle atrophy, Squelettal muscle paralysis, Cognitive or behavioral dysfunction	Riluzole

**Table 2:** Recently reported curcumin (CU) activities in in vitro and in vivo models of neurodegenerative diseases [41,42,43,44,45,46,47,48,49,50].

Disease	Model/Administration Route	Mechanism	Outcomes
AD	In vitro: human neuroblastoma SH-SY5Y and IMR-32 cells	Enhancement of the expression of DNA repair enzymes (APE1, pol β, and PARP1 ^1^) to halt the oxidative DNA base damage via base excision repair (BER) pathway; Activation of the antioxidant response element (ARE) via Nrf2 upregulation	Revitalization of the neuronal cells from Aβ ^2^ induced oxidative stress [41].
AD	In vitro: mouse hippocampal clone neuronal cell line HT-22 cells treated with Aβ 1-42, In vivo: mice with APP/PS1 transgenes	Decrease of the autophagosomes number, Increase of the lysosomal Ca^2+^ regulation of PI(3,5)P2 and Transient Receptor Potential Mucolipin-1 Expression (TRPME)	Neuronal cell growth, Protective role of CU on memory and cognition impairments [42].
AD	In vivo: rat, oral supplementation	Increase of GPx ^3^, CAT ^4^, GSH ^5^ activities and Ach ^6^ levels	Improving memory and cognitive abilities [43].
PD	In vivo: *Drosophila* model of PD with dUCH ^7^ knockdown	Effects on dUCH ^7^ knockdown, a homolog of human UCH-L1	Decrease of ROS levels, Improved locomotive abilities, Reduction of dopaminergic neurons degeneration [44].
PD	In vivo: male Sprague-Dawley rats injured by 6-OHDA ^8^ in the left striatum	Activation of the Wnt/β-catenin signaling pathway, Higher Wnt3a and β-catenin mRNA and protein expressions, c-myc and cyclin D1 mRNA expression, enhanced SOD ^9^ and GPx ^3^ contents, decreased MDA ^10^ content and elevated mitochondrial membrane potential	Protective effect of CU against oxidative stress-induced injury, Enhanced viability, survival, and adhesion, attenuated apoptosis of deutocerebrum primary cells [45].
PD	In vivo: MPTP ^11^ mice, intranasal mode of administration of CU (mucoadhesive system)	Increase of dopamine concentration in brain, which improves muscular coordination and gross behavioral activities of the test animal, Significant reduction of the MPTP^11^-mediated dopamine depletion	Improvement in motor performance, Symptomatic neuroprotection against MPTP-induced neurodegeneration in the striatum [46].
HD	In vivo: CAG140 mice, a knock-in (KI) mouse model of HD	Decreased huntingtin aggregates, increased striatal DARPP-32 and D1 receptor mRNAs	Partial improvement of transcriptional deficits, partial behavioral improvement [47].
Diazepam-induced cognitive impairment	In vivo: diazepam-treated rats, oral supplementation	Downregulation of the extracellular signal-regulated kinase (ERK 1/2)/nuclear transcription factor-(NF-)*κ*B/pNF-*κ*B pathway in the hippocampus and the iNOS ^12^ expression in the hippocampus and frontal cortex	Improvement of the cognitive performance, Decrease of blood and brain oxidative stress levels [48].
Alcohol-induced neurodege neration	In vivo: rat, oral supplementation	Decrease of the reduced form of GSH ^5^, SOD ^9^, GPx ^3^, GR ^13^, change in the Bcl-2 levels, Activation of the CREB-BDNF signaling pathway	Neuroprotection against alcohol-induced oxidative stress, apoptosis and inflammation [49].
Nicotine-induced neurodege neration	In vivo: rat, oral supplementation	Activation of the CREB-BDNF signaling pathway	Neuroprotection against nicotine-induced inflammation, apoptosis and oxidative stress, Reduction of the motor activity disturbances [50].

^1^ Poly [ADP-ribose] polymerase 1; ^2^ Aβ-amyloid; ^3^ Glutathione Peroxidase; ^4^ Catalase; ^5^ Glutathione; ^6^ Acetylcholine; ^7^ Ubiquitin carboxy-terminal hydrolase; ^8^ 6-Hydroxydopamine; ^9^ Superoxide dismutase; ^10^ Malondialdehyde; ^11^ 1-methyl-4-phenyl-1,2,3,6-tetrahydropyridine; ^12^ Induced Nitric Oxide Synthase; ^13^ Glutathione Reductase.

**Table 3:** Curcumin-loaded polymeric nanoparticles studied in in vitro and in vivo models of neurodegenerative diseases.

Disease	Nanoformulation Type	Model/Administration Route	Outcomes
AD	PLGA ^1^ nanoparticles	In vitro: SK-N-SH human neuroblastoma cells	Protection against H_2_O_2_-induced oxidative damage [70].
AD	PLGA nanoparticles	In vitro: Neural stem cells, In vivo: Aβ ^2^-amyloid induced rat model of AD-like phenotypes	Expression of genes involved in neuronal proliferation and differentiation, Reverse learning and memory impairments [73].
AD	PLGA nanoparticles conjugated with Tet-1 peptide	In vitro	Anti-amyloid activity unchanged, decrease of aggregates size [74], Diminution of anti-oxidant activity.
AD	PLGA nanoparticles functionalized with glutathione	In vitro: in SK-N-SH cells	Neuronal uptake, Enhanced curcumin action [75,76].
AD	PLGA nanoparticles	In vivo: Rat, IV, oral	Increased CU bioavailability and plasma concentration [77].
AD	PLGA nanoparticles	In vivo: Rat	Prolonged CU retention time in cerebral cortex and hippocampus [78].
AD	Apolipoprotein E3-mediated poly(butyl)cyano acrylate nanoparticles	In vitro: SH-SY5Y cells	Protection against Aβ-induced cytotoxicity [79].
AD	Pegylated poly(alkyl cyanoacrylate) nanoparticles with anti-Aβ 1–42 antibody at the surface	In vitro	Inhibition of Aβ aggregation [80].
AD	Spherical (SPNs) or Discoidal (DPNs) polymeric nanocontructs PLGA, DSPE-PEG ^3^	In vitro: Raw 264.7 cells In vitro production of Aβ fibers	Decrease of the pro-inflammatory cytokines in macrophages stimulated via Aβ fibers [81]
AD	Polymeric nanoparticles (NanoCurc^TM^)	In vitro: SK-N-SH differentiated cells In vivo: Mice, parenteral injection	Protection against H_2_O_2_-induced oxidative stress, Downregulation of caspase 3 and 7 activities, mediators of the apoptotic pathway, Increased glutathione levels [82].
AD	Nanocurcumin CU within polyethylene glycol-polylactide diblock polymer micelles	In vitro In vivo: AD model Tg2576 mice	Higher curcumin concentration in plasma, 6 times higher area under the curve and mean residence time in brain than ordinary CU, Improved memory function [83].
AD	Nanoemulsion	In vitro: SK-N-SH cell line, Sheep nasal mucosa	Safe for intranasal delivery for brain targeting, Higher flux and permeation across sheep nasal mucosa [84].
Mitochon drial dysfunction in brain aging	Micelles	In vitro: PC12 cells In vivo: NMRI mice; Ex vivo: isolated mouse brain mitochondria	Improved bioavailability of native curcumin around 10- to 40-fold in plasma and brain of mice, Prevention of mitochondrial swelling in isolated mouse brain mitochondria, Protection of PC12 cells from nitrosative stress as compared to free CU [85].
PD	Alginate nanocomposites	In vivo: Drosophila, oral	Reduction of oxidative stress and apoptosis in the brain [86].

^1^ Poly(lactic-co-glycolic acid); ^2^ Aβ-amyloid; ^3^ Distearoy phosphatidylethanolamine-Polyethylene glycol.

**Table 4:** Curcumin-loaded lipid nanoparticles studied in in vitro and in vivo models of neurodegenerative diseases.

Disease	Nanoformulation Type	Model/Administration Route	Outcomes
AD	Solid lipid nanoparticles	In vitro: Mouse neuroblastoma cells after Aβ ^1^ exposure	Decreased ROS production, Prevented apoptotic death, Inhibition of Tau formation [89,90].
AD	Solid lipid curcumin particle (SLCP), Longvida^®^	In vitro: lipopolysaccharide (LPS)-stimulated RAW 264.7 cultured murine macrophages.	Improved solubility over unformulated curcumin, Decreased LPS induced pro-inflammatory mediators NO, PGE2, and IL-6 by inhibiting the activation of NF-kB [92].
AD	Solid lipid particleswith CU (SLCP)	In vivo: one-year-old 5xFAD-and age-matched wild-type mice, intraperitoneal injections of CU/SLCP	Decrease in Aβ plaque loads in dentate gyrus of hippocampus, More anti-amyloid, anti-inflammatory, and neuroprotective [91].
AD	Solid lipid nanoparticles	In vivo: Rat, oral	Effective delivery across the BBB ^2^ [88].
HD	Solid lipid nanoparticles (CU-SLNs)	In vivo: (3-NP)-induced HD in rats	Restored glutathione levels and superoxide dismutase activity, Activation of nuclear factor-erythroid 2 antioxidant pathway, Reduction in mitochondrial swelling, lipid peroxidation, protein carbonyls and reactive oxygen species [89].
CNS disorders	Solid lipid nanoparticles (CU-SLNs) and nanostructured lipid carriers (CU-NLCs)	In vivo: male Sprague−Dawley rats 6−8 weeks old, oral	Enhanced curcumin brain uptake, Cur-NLCs enhance the absorption of brain curcumin more than 4-folds in comparison with Cur-SLNs [95].
AD	Lipoprotein (LDL)-mimic nanostructured lipid carrier (NLC) modified with lactoferrin (Lf) and loaded with CU	In vivo: Rat, oral	Cellular uptake mediated by the Lf receptor, Permeability through the BBB and preferentially accumulation in the brain, Efficacy in controlling the damage associated with AD [96].
AD	Liposomes functionalized with TAT-peptide	In vitro	Permeability across the BBB enhanced [98].
AD	Liposomes containing cardiolipin	In vitro: SK-N-MC cells	Inhibition of the phosphorylation of p38, JNK, and tau protein, Protection against serious degeneration of Aβ insulted neurons [101].
AD	WGA ^3^-conjugated and cardiolipin-incorporated liposomes carrying NGF ^4^ and CU	In vitro: Human astrocytes and to protect SK-N-MC cells Apoptosis induced by β-amyloid1–42 (Aβ 1–42) fibrils	Increased entrapment efficiency of NGF and CU, of NGF release and cell viability, Decreased release of CU, Permeability of NGF and CU across the blood–brain barrier [102].
AD	Liposomes	In vivo: Mice, stereotaxic injection in the right hippocampus and neocortex	Decrease in Aβ secretion and toxicity [97].
AD	Liposomes decorated with anti-transferrin receptor mAb	In vivo injection, hippocampus and neocortex	Decrease in Aβ 1–42 aggregation, Internalization in the BBB model [99].
AD	Liposomes functionalized with a curcumin-alkyne derivative TREG	Human biological fluids from sporadic AD patients and down syndrome subjects	Sequestration of Aβ 1–42 [100,101].
Neuronal loss	Liquid-crystalline lipid nanoparticles carrying curcumin and DHA	In vitro: SH-SY5Y cells	Neuronal viability and neurite outgrowth by activation of the TrkB receptor signaling, and promotion of phosphorylated CREB protein expression [118].
AD	Lipopeptide: a short microtubule- stabilizing peptide conjugated to a hydrophobic palmitic acid chain	In vitro: Neuro-2a cells, PC-12 differentiated cells	Neurite outgrowth in absence of external growth factors, Neural cells morphology and health amelioration [120,121].

^1^ Aβ-amyloid; ^2^ Blood-brain barrier; ^3^ Wheat germ agglutinins; ^4^ Nerve growth factor.

## Abbreviations

Aβ	Amyloid βeta
ACh	Acetylcholine
AD	Alzheimer’s disease
ALS	Amyotrophic lateral sclerosis
ApoE	Apolipoprotein E
APP	Amyloid beta precursor protein
ARE	Antioxidant response element
BBB	Brain blood barrier
BDNF	Brain derived neurotrophic factor
Ca^2+^	Calcium ion
CAT	Catalase
CHCHD2	Coiled-coil-helix-coiled-coil-helix domain 2
C9ORF72	Chromosome 9 open reading frame 72
COMT	Catechol-*O*-methyltransferase
CREB	cAMP (Cyclic adenosine monophosphate response) element-binding protein
CU	Curcumin
CYP	Cytochrome P450
DARPP	Dopamine and adenosine 3′,5′-monophosphate-regulated phosphoprotein
DHA	Docosahexaenoic acid
DNA	Deoxyribonucleic acid acid
DNAJC13	DNA J heat shock protein family (Hsp40) member C13
DSPE	Distearoy phosphatidylethanolamine
EIF4G1	Eukaryotic translation initiation factor 4 gamma 1
EMA	European medicines agency
ERK	Extracellular signal regulated kinase
FDA	Food and drug administration
FUS	RNA binding protein Fused in Sarcoma
GBA	Glucocerebrosidase
GRAS	Generally recognized as safe
GPx	Glutathione peroxidase
GR	Glutathione reductase
GSH	Glutathione
HEWL	Hen Egg White Lysozyme
HD	Huntington disease
H_2_O_2_	Hydrogen peroxide
HTT	Huntingtin
IL-6	Interleukin 6
iNOS	induced nitric oxide synthase
IV	intravenous
JNK	Jun N-terminal kinase
LCNs	Liquid crystalline nanocarriers
LDL	Low density lipoprotein
Lf	Lactoferrin
LPS	Lipopolysaccharide
LRRK1	Leucine-rich repeat kinase 1
LUV	Large unilamellar vesicles
MAO-B	Monoamine oxidase type B
MDA	Malondialdehyde
MLV	Multilamellar vesicles
MPTP	1-methyl-4-phenyl-1,2,3,6-tetrahydropyridine
MMSE	Mini Mental State Examination
mRNA	Messenger Ribonucleic Acid
NF-kb	Nuclear Factor Kappa Beta
NGF	Nerve growth factor
NLC	Nanostructured lipid carriers
NO	Nitric oxide
NPs	nanoparticles
Nrf2	Nuclear factor erythroid 2–related factor 2
NSAIDs	Non-steroidal anti-inflammatory drugs
OHDA	6 Hydroxydopamine
OPTN	Optineurin
PD	Parkinson disease
PEG	Polyethylene glycol
PINK1	PTEN-induced putative kinase 1
PLGA	Poly (lactic-*co*-glycolic acid)
PRKN	Parkin
PSEN	Presenilin
PUFA	Polyunsaturated fatty acids
ROS	Reactive oxygen species
SLCP	Solid lipid curcumin nanoparticles
SLN	Solid lipid nanoparticles
SOD1	Superoxide dismutase 1
SNCA	Synuclein alpha
SUV	Small unilamellar vesicles
TARDBP	TAR DNA binding protein (TDP-43)
TREG	T regulatory cell
TRPME	Transient Receptor Potential Mucolipin-1 Expression
TrkB	Tropomyosin receptor kinase B
UBQLN2	Ubiquitin 2
UCH	Ubiquitin carboxy-terminal hydrolase
VPS35	Vascular protein sorting
WGA	Wheat-germ agglutinin
